# Early Renal Histological Changes in Alloxan-Induced Diabetic Rats

**Published:** 2014

**Authors:** Mohsen Pourghasem, Ebrahim Nasiri, Hamid Shafi

**Affiliations:** 1 *Cellular and Molecular Biology Research Center (CMBRC), Babol University of Medical Sciences, Babol, Iran.*; 2 *Department of Anatomical Sciences, Gilan University of Medical Sciences, Rasht, Iran.*; 3 *Department of Urology, Babol University of Medical Sciences, Babol, Iran.*

**Keywords:** Diabetes mellitus, kidney, alloxan

## Abstract

Diabetes mellitus is a progressive disease. Most investigators have focused on glomerular changes in diabetic kidney and non-glomerular alterations have been less attended. The present study has been conducted to find early non-glomerular histological changes in diabetic renal tissue. Twenty male Wistar rats weighting 200-250 g were used for the diabetic group. Diabetes mellitus was induced by single injection of Alloxan. After 8 weeks, paraffin embedded blocks of kidneys were prepared for evaluating the histological changes due to diabetes. Histological study showed the deposit of eosinophilic materials in the intermediate substantial of medulla and thickening of renal arterial wall in the kidney of 70% of diabetic rats. The average weight of kidneys increased when compared to non diabetic animals. Furthermore, the amount of blood flow in arteries of all diabetic kidneys has been enhanced. The present study demonstrates some early renal histological changes in diabetes mellitus which were earlier compared to those reported previously. Diabetic nephropathy is a progressive disease and renal care design can help better prognosis achievement.

Diabetes mellitus is the most common cause of chronic renal disorders and end stage kidney disease in developed countries. It is the major cause of dialysis and transplantation. The development of diabetic nephropathy is associated to several factors such as genetic susceptibility, hemodynamic and biochemical changes (1). All sizes of arteries can be affected in the diabetes mellitus (2). Therefore, both micro and macro angiopathy can be seen in the diabetic kidney. The pathogenesis of diabetic nephropathy is associated with the duration and efficiency of treatment of hyperglycemia and blood pressure in diabetes mellitus (3-4).

Most investigations have shown that the earliest detectable changes in the course of diabetic nephropathy in human will be seen 10 years after diabetes mellitus initiation. However, morpho-metric studies showed that the signs can be diagnosed 18 months after diabetes initiation (5). Diabetic nephropathy is a progressive disease and earlier diagnosis can help in making a better treatment design in order to reduce its development. For example, using sulodexide regulates matrix protein accumulation in diabetic nephropathy (6). Most researches are investigating glomerular alterations due to diabetes and the effects of non-glomerular structure changes on clinical spectrum of diabetic nephropathy have been much less attended.

A wide range of alterations in the renal tissue have been described in diabetes. Renal tubular function has been changed and consequently there are tubular proteinuria, sodium and glucose transport disorders (7-8).

In our previous researches, we had shown an increase of lipofuscin pigments in the renal tubular cells and increased glomerular mesangium at an earlier time in the kidney of a diabetic rat (9-10). This research was conducted to find the very early renal histological changes in the diabetes mellitus.

## Materials and Methods

Based on an experimental study, 20 male Wistar rats (weight 200 - 250 g) were considered as experimental group. Weight and time-matched rats were used as control animals. The animals were housed in a standard laboratory condition, 12 hours light/darkness cycle, constant temperature, 50-55% moisture and easy access to food and water. Animal care was performed in accordance with the Ethics Committee of Babol University of Medical Sciences. Diabetes mellitus was induced by a single subcutaneous injection (120 mg/kg) of freshly prepared solution of alloxan monohydrate (Aldrich, A7413-25G) dissolved in PBS subcutaneously (11). The induction of hyperglycemia was confirmed one week after treatment and the day of sacrificing by blood glucometer (Gluco care.77 Electronica kft, co) in rats with fasting blood glucose levels above 200 mg/dl. Five rats were excluded due to blood glucose levels lower than 200 mg/dl and/ or death. At the end of the 8th week of treatment, both the control and experimental groups were anesthetized with pentobarbital (80 mg/kg, IP) before perfusion through the heart with 10% formaldehyde. Right kidneys were dissected and rinsed in cold saline. After weighing, the kidneys were immersed in 10% formaldehyde for 48 h and then were paraffin embedded and sectioned at 5μm on a microtome. The slides were prepared and studied after being stained with Hematoxilin – Eosin (H&E) using a light microscope (Olympus, Tokyo, Japan). For the comparison of kidney weight between the control and experimental groups, T test was performed and p-value < 0.05 was considered as statistically significant.

## Results

**Fig 1:**
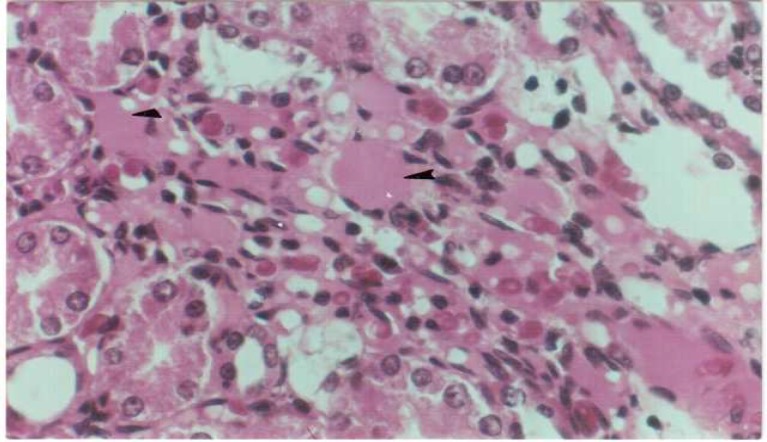
Eosinophilic deposits in the diabetic kidney (Arrow). H&E × 400

Histological study showed a deposit of eosinophilic materials in the intermediate substantial of medulla in the kidney of 73% (11 rats) of diabetic animals (Figure 1). Vacuolar changes have been seen in the tubular cells of all diabetic kidneys. The average weight of kidneys increased compared to nondiabetic animals (P< 0.05, table 1).

**Table 1:** kidney weight in control and diabetic groups

Weight range (gram)	Diabetic Control n (%) n (%)	
0.77–0.87	0	3(20%)
0.88–0.98	0	10(67%)
0.99–1.09	2(13%)	2(13%)
1.1–1.2	9(60%)	0
1.21–1.31	4(27%)	0
Total	15(100%)	15(100%)

**Fig 2:**
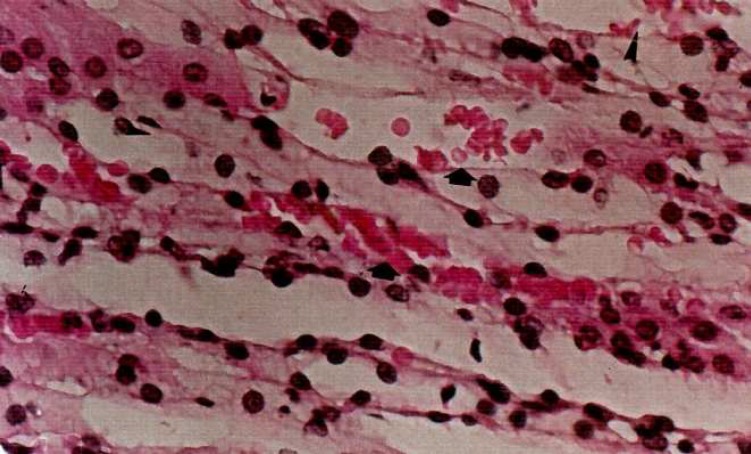
Increasing of blood flow in the diabetic rats. H&E × 400

The amount of blood flow in arteries of all diabetic kidneys especially in vasa recta have been enhanced (Figure 2).

**Fig 3:**
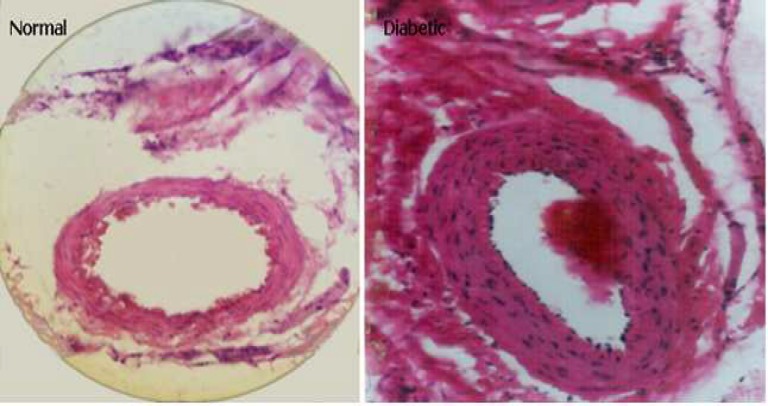
Increased thickness of renal artery in the diabetic kidney

Compared to matched renal arterial lumen diameter of control group, the increase of thickness in the intrarenal arterial walls has been observed in all diabetic rats (Figure 3). 

## Discussion

This study has been conducted to find earlier possible histological changes in the kidney of diabetic rats. Eight weeks after the initiation of diabetes mellitus, the histological study of kidney demonstrated the presence of abnormal cells in the wall of renal tubules which could lead to cell damages. The cytoplasm resolution of abnormal cells changed and vacuolar modifications occurred. According to the association between cell shape and cell function, these changes may correspond to an adaptation of cells to a new situation such as increased load. This is in agreement with an increase of lipofuscin pigments in the renal tubular cells which has been reported previously (10). The increase of lipofuscin pigments may represent an over stress status of cells. Vacuolar changes may correspond to initiation of Armanni – Ebstein lesion associated to glycogen deposition or subnuclear lipid vacuolization. It can be reduced by an anti-fibrotic and anti-inflammatory agent (12). The deposition of eosinophilic materials which is presented in this study can be an early sign of kidney affection occurring in diabetes mellitus. Eosinophilic materials display and represent accumulated materials in the interstitial space. This may be an early sign of renal fibrosis. Tubulointerstitial fibrosis originates from non-vascular injury and thus, can represent an imbalance between the synthesis and degradation of extra cellular matrix (ECM) which occurs upon glycation. The event has been reported by Sugimotto et al. but 6 months after diabetes initiation (13). The glycation of ECM proteins changes both their structure and function.

Glycation is a nonenzymatic reaction between sugars and the free amino groups of materials in a hyperglycemic situation such as diabetes mellitus. The glycation of materials induces a wide range of chemical, cellular and tissue effects and leads to nephropathy development. Sabbatini et al. showed that the early glycation products (EGPs) induce glomerular hyperfiltration even in normal rats (14). The glycation process is reversible but over time, it becomes irreversible and EGPs develop into advanced glycation end products (AGEs). AGE influences charge, solubility and conformation of ECM. Therefore, the early diagnosis and treatment of hyperglycemia prevents AGE production.

Renal investigations have demonstrated that hyperfiltration is associated to vasodilatation and the consequent increase in blood flow and glomerular capillary pressure (15-16). The present study made obvious the blood flow amplification in the diabetic kidney. The blood flow increase is the primary and main cause of structural and functional disorders in the kidney and blood flow correction in the early stage of diabetes inhibits further complications in the kidney (17).

The kidney weight increased when compared to control animals. This result indicates that the onset of renal enlargement can be a characteristic feature of diabetic kidney. It has manifested that the kidney enlargement is caused by certain factors like glucose over-administration, glycogen accu-mulation, lipogenesis and protein synthesis in the diabetic kidney (18-19). Actually, it is due to glomerular hypertrophy and nephromegaly. To compare with our study, Kiran et al. reported that the increase of kidney weight initiated from the first month of diabetes mellitus and is exaggerated at the end of the fourth month which is even earlier compared to the present study (20). Kidney enlargemement can be easily diagnosed by a noninvasive method such as ultrasonography.

Vascular hypertrophy is presented in this study. The thickening of renal arterial wall during 8 weeks diabetes needs more attention. The result is in agreement with those, have been reported previously (21-22). It is a progressive complication and leads to hypertension and ischemic nephropathy. Increased angiotensin II due to diabetes mellitus is the most important cause of arterial wall hypertrophy and atherosclerosis. It stimulates proliferation of smooth and mesangial cells (23-24). Thus, it is now known that angiotensin converting enzyme inhibitor can reduce and correct diabetic nephropathy (25). Sato et al. showed the structural changes of renal arteries that are already prominent before glomerular changes and advanced kidney disorders (25).

In conclusion, there are many early nonglomerular structural changes in the diabetic kidney which need more attention for patient care. Furthermore, the diagnosis of affected kidney in diabetes is possible before prominent functional disorders occur.
